# Causal linkage between adult height and kidney function: An integrated population-scale observational analysis and Mendelian randomization study

**DOI:** 10.1371/journal.pone.0254649

**Published:** 2021-07-29

**Authors:** Sehoon Park, Soojin Lee, Yaerim Kim, Yeonhee Lee, Min Woo Kang, Kwangsoo Kim, Yong Chul Kim, Seung Seok Han, Hajeong Lee, Jung Pyo Lee, Kwon Wook Joo, Chun Soo Lim, Yon Su Kim, Dong Ki Kim

**Affiliations:** 1 Department of Biomedical Sciences, Seoul National University College of Medicine, Seoul, Korea; 2 Department of Internal Medicine, Armed Forces Capital Hospital, Gyeonggi-do, Korea; 3 Division of Nephrology, Department of Internal Medicine, Uijeongbu Eulji University Medical Center, Gyeonggi-do, Korea; 4 Department of Internal Medicine, Keimyung University School of Medicine, Daegu, Korea; 5 Department of Internal Medicine, Seoul National University Hospital, Seoul, Korea; 6 Transdisciplinary Department of Medicine & Advanced Technology, Seoul National University Hospital, Seoul, Korea; 7 Kidney Research Institute, Seoul National University, Seoul, Korea; 8 Department of Internal Medicine, Seoul National University College of Medicine, Seoul, Korea; 9 Department of Internal Medicine, SMG-SNU Boramae Medical Center, Seoul, Korea; Yale University School of Medicine, UNITED STATES

## Abstract

As adult height is linked to various health outcomes, further investigation of its causal effects on kidney function later in life is warranted. This study involved a cross-sectional observational analysis and summary-level Mendelian randomization (MR) analysis. First, the observational association between height and estimated GFR determined by creatinine (eGFR_creatinine_) or cystatin C (eGFR_cystatinC_) was investigated in 467,182 individuals aged 40–69 using UK Biobank. Second, the genetic instrument for adult height, as reported by the GIANT consortium, was implemented, and summary-level MR of eGFR_creatinine_ and CKD_creatinine_ in a CKDGen genome-wide association study was performed (N = 567,460), with multivariable MR being adjusted for the effects of genetic predisposition on body mass index. To replicate the findings, additional two-sample MR using the summary statistics of eGFR_cystatinC_ and CKD_cystatinC_ in UK Biobank was performed (N = 321,405). In observational analysis, adult height was inversely associated with both eGFR_creatinine_ (per 1 SD, adjusted beta -1.039, standard error 0.129, P < 0.001) and eGFR_cystatinC_ (adjusted beta -1.769, standard error 0.161, P < 0.001) in a multivariable model adjusted for clinicodemographic, anthropometric, metabolic, and social factors. Moreover, multivariable summary-level MR showed that a taller genetically predicted adult height was causally linked to a lower log-eGFR_creatinine_ (adjusted beta -0.007, standard error 0.001, P < 0.001) and a higher risk of CKD_creatinine_ (adjusted beta 0.083, standard error 0.019, P < 0.001). Other pleiotropy-robust sensitivity MR analysis results supported the findings. In addition, similar results were obtained by two-sample MR of eGFR_cystatinC_ (adjusted beta -1.303, standard error 0.140, P < 0.001) and CKD_cystatinC_ (adjusted beta 0.153, standard error 0.025, P < 0.001) in UK Biobank. In conclusion, the results of this study suggest that a taller adult height is causally linked to worse kidney function in middle-aged to elderly individuals, independent of the effect of body mass index.

## Introduction

Kidney disease is a major category of comorbidity, and the prevalence of chronic kidney disease (CKD) is increasing with the aging population and global obesity epidemic [1]. Impaired kidney function negatively affects quality of life and various disorders. Recent evidence suggests that an estimated 5–10 million people die due to kidney disease each year [2]. Thus, identifying various factors affecting kidney function is important.

Kidney function is commonly evaluated by estimated values, namely, the estimated glomerular filtration rate (eGFR), due to its greater availability than a directly measured GFR. Because of widespread serum creatinine testing and robustly developed eGFR equations [3, 4], eGFR is often determined in routine health exams or when assessing the general medical condition of a patient. Lower eGFR, indicating a kidney function decline, is a sensitive biomarker related to risks of various health outcomes. Furthermore, considering that serum creatinine has a limitation in that it can be affected by body mass or dietary factors, the advantage of using a cystatin C-based eGFR, which is less affected by such bias, has been reported [4, 5].

Height is an important factor related to health outcomes [6], as is body mass index (BMI) which has been the primary focus of modern medicine regarding obesity issues. Common variants explain the majority (60%) of the genetic heritability in height. Previous studies have reported that taller height may confer a lower risk of coronary artery disease and a better socioeconomic status based on Mendelian randomization (MR) analysis [7–9], which can reveal causal effects of exposure traits on the occurrence of complex diseases. However, the causal effect of height, independent of BMI, on kidney function has rarely been studied. Nevertheless, a previous study showed that a taller height is related to a higher single-nephron GFR in individuals with normal kidney function, similar to obesity, suggesting that a taller height is a potential risk factor for glomerular overload related to consequent kidney function impairment in later life [10]. Additionally, a recent study identified a larger glomerular size, as in those with obesity or hypertension, in taller individuals [11]. On the other hand, a possibility remains that biomarkers for determining eGFR may be affected by height, resulting in inappropriately estimated kidney function [12]. Thus, further investigation is necessary to examine the association and causal linkage between adult height and kidney function in middle-aged to elderly individuals.

In the abovementioned MR analysis, genetic instruments were used to examine the association between genetically predicted exposure and outcome traits [13]. As one’s genotype is determined before birth, the genetically proxied phenotype is less likely affected by confounding effects or reverse causation. Therefore, MR analysis is widely adopted in the medical literature to investigate causative pathways between complex exposure and outcome traits, also benefiting from the recent availability of large-scale genetic data [14]. MR analysis has also been recently implemented in the field of nephrology to reveal risk factors for CKD [15–20].

Here, we present a cross-sectional observational study and an MR analysis focusing on causal effects of adult height on eGFR in middle-aged to elderly individuals. Along with our recent efforts to identify causative factors for kidney function through MR investigations [15–18], we hypothesized that a taller adult height, as adjusted for the effects of BMI, would be causally linked to lower kidney function in the middle-aged to elderly population.

## Materials and methods

### Ethics approval

The study was performed in accordance with the Declaration of Helsinki. The study was approved by the institutional review boards of Seoul National University Hospital (No. E-2005-006-1120) and the UK Biobank consortium (application No. 53799). Informed consent was waived as the study analyzed anonymous databases.

### Study setting

The study incorporated three databases or their findings: 1) the previous GIANT consortium genome-wide association study (GWAS) meta-analysis for height, which was implemented to identify genetic instruments for height; 2) the previous CKDGen consortium GWAS for kidney function traits based on serum creatinine values, which was implemented as the outcome summary statistics for summary-level MR; and 3) UK Biobank data, which were used for cross-sectional observational analysis. The UK Biobank data was also used as another summary statistics for kidney function traits based on cystatin C levels for summary-level MR.

**Fig 1:**
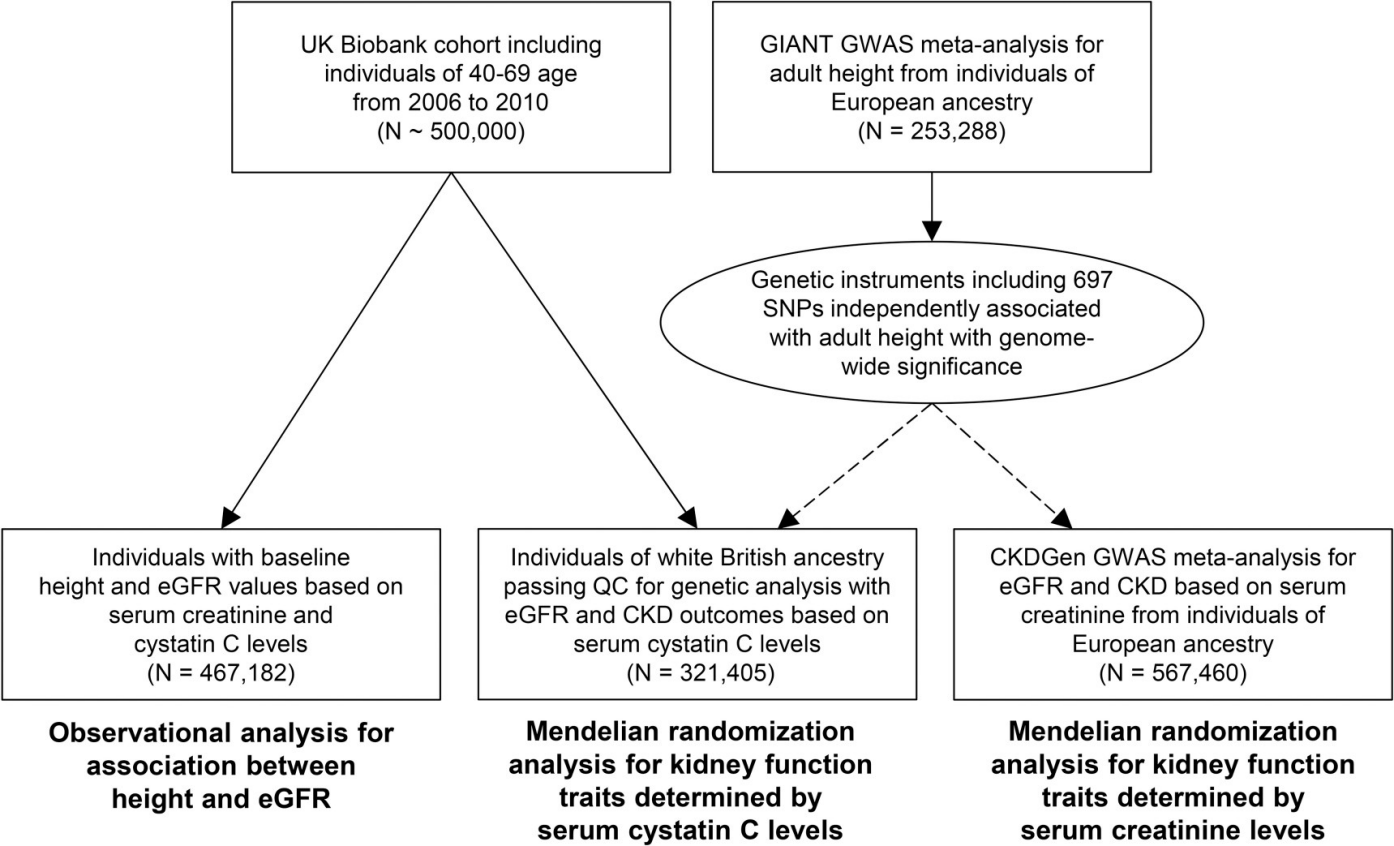
Study flow diagram. CKD = chronic kidney disease, eGFR = estimated glomerular filtration rate, GWAS = genome-wide association study, SNP = single-nucleotide polymorphism, QC = quality control, MR = Mendelian randomization.

The study mainly consisted of two parts (Fig 1). First, cross-sectional observational analysis was performed to investigate the association between adult height and eGFR measured in the middle-aged to elderly population in UK Biobank. However, as the investigation was cross-sectional and observational, the possibility of unmeasured confounding effects or reverse causation existed, whereby the results would be rather supportive and causal inference limited. Therefore, we further performed summary-level MR analyses for the eGFR or CKD outcomes using a genetic instrument for adult height developed from the GIANT study. UK Biobank data and other summary statistics for kidney function traits by the CKDGen consortium were utilized as outcome information of the summary-level MR [21].

### Cross-sectional observational analysis

The abovementioned cross-sectional observational analysis was performed with UK Biobank data. In brief, UK Biobank is a population-scale prospective cohort including participants aged 40–69. The study recruited > 500,000 participants from 2006 to 2010 from 22 assessment centers in the United Kingdom, and the study is powered by its extensively phenotyped and genotyped information. Other details of the UK Biobank project can be found in the literature [22, 23].

For the cross-sectional observational study, we included 467,182 UK Biobank participants with adult height values, as measured by assessment staff during the inclusion visit, and both eGFR (creatinine) and eGFR (cystatin C), as calculated by the CKD-EPI equation, which includes information on biomarkers, age, sex, and ethnicity [3, 4]. The serum creatinine and cystatin C values reported in UK Biobank were measured using standardized enzymatic methods, and a previous study found that eGFR (cystatin C) is less affected by body shape measures or diet and showed superior value when predicting patient-oriented outcomes [5]. In the current observational analysis, we investigated both eGFR (creatinine) and eGFR (cystatin C) measured during the baseline visits as outcome variables.

First, the overall association between adult height and eGFR values was investigated by generalized additive models adjusted for age and BMI for each sex. Second, linear regression analysis was performed with the construction of stepwise multivariable models. The first model included basic factors, age, sex, and BMI. The second multivariable model included a list of body index variables, adding waist circumference, weight, and whole-body fat-free mass, as measured by a BC418 MA body composition analyzer (Tanita, Tokyo, Japan). The third multivariable model included a wide range of metabolic parameters and social factors collected at inclusion visits, such as hypertension history, systolic BP, diastolic BP, dyslipidemia medication, LDL cholesterol, HDL cholesterol, triglycerides, diabetes mellitus, hemoglobin A1c, testosterone level, uric acid level, history of angina/heart attack/stroke, history of cancer, average moderate physical activity frequency, and smoking history. As information was missing in the dataset, the third fully adjusted model was repeatedly constructed in an imputed dataset established by multiple imputation with the changed equation method [24]. The analysis was performed with the “glm” command in R (version 3.6.2, the R Foundation). The details of the covariates collected and missing information are described in S1 Methods.

### Concept of MR and MR assumptions

As causal inference by observational analysis is limited, we further extended our findings to an MR investigation. In MR, genotype-determined inborn individuals are used as genetic instruments for an exposure trait of interest, and the association between genetically predicted exposure and an outcome trait is tested. Genetically proxied exposure can less likely be affected by confounders, and the direction of the association is toward outcome; thus, a significant finding in MR can support causal effects of the exposure trait on the outcome.

A valid MR study requires three assumptions to be met to suggest causal inference [13]. First, the relevance assumption is that the genetic instrument should be closely associated with the exposure phenotype of interest. As the genetic instrument explained a certain variance regarding height and was reported in a large-scale GWAS, this assumption was considered to have been met. Particular caution is necessary with regard to the other two assumptions, i.e., exclusion-restriction and independence assumptions, meaning the absence of a horizontal pleiotropic pathway. The independence assumption is that the instrument should not be closely associated with confounding phenotypes. We performed various pleiotropy-robust MR sensitivity analyses to support attainment of the assumption and calculated the MR-Egger intercept P value, which is used to identify the presence of a significant pleiotropic effect. In addition, we performed sensitivity analysis by excluding single-nucleotide polymorphisms (SNPs) strongly associated with potential confounders. Although the exclusion restriction assumption is yet untestable, some pleiotropy-robust MR analyses relax this assumption in a portion of the instruments, further supporting the main causal estimates.

### Genetic instruments for height exposure

We used the genetic instrument for adult height reported by the GIANT consortium [25], and the study was a GWAS meta-analysis of adult height using summary statistics from 79 GWASs involving 253,288 individuals of European ancestry. The study reported 697 independent SNPs reaching the genome-wide significance level (P < 5×10^−8^) for height by conditional and joint association analysis. Using the SNPs reported in the study has particular strength for an MR analysis as the samples included in the GWAS meta-analysis were completely independent from the UK Biobank data, allowing a two-sample MR analysis. Two-sample MR analysis has strength because the possibility of false-positive findings is minimal, supporting the robustness of a positive finding in MR, and such two-sample approach would not be possible if we used a different, recently reported GWAS results [26] as there were overlapping samples.

The previously reported 697 SNPs associated with adult height used in this study had explained variance of 15.9%. We first included all 697 SNPs in our investigations (S2, S3, S4, S5 Tables); we also performed further sensitivity analysis excluding SNPs with potential associations with possible confounders to robustly attain the independence assumption (S6 Table).

### Outcome summary statistics for summary-level MR

As mentioned above, first, we implemented the recent CKDGen GWAS meta-analysis for log-transformed eGFR based on creatinine and CKD [creatinine-based eGFR < 60 mL/min/1.73 m^2^] for 567,460 individuals of European ancestry [21]. These data are the largest scale summary statistics for kidney function traits, and the study reported 308 index SNPs explaining 7.1% eGFR variance and 19.6% eGFR genetic heritability. The individuals of European ancestry in CKDGen had a median age of 54 years, and 50% of them were male, which suggests that the eGFR values were obtained from a generally middle-aged population. The CKDGen data had partial overlap with the samples included in the GIANT study (e.g., TWINGENE). Summary-level MR was performed with the genetic instrument for the summary statistics of kidney function outcomes.

Because creatinine values are likely to be affected by various body shape factors, we produced summary statistics for continuous eGFR based on the cystatin C level and CKD (eGFR < 60 mL/min/1.73 m^2^ or prevalent end-stage kidney disease history) using UK Biobank data, which is independent from the samples involved in the genetic instrument identification [4]. We identified 337,138 individuals of white British ancestry passing the basic quality control process; there were no cases of excess kinship, sex chromosome aneuploidy, or outliers in terms of heterozygosity or missing rate. The detailed genotyping and imputation process for the UK Biobank genetic data has been previously described [14]. Among the participants, baseline eGFR (cystatin C) values were available for 321,405 and were included for generating outcome summary statistics. Linear regression or logistic regression for the eGFR (cystatin C) and CKD (cystatin C) model was constructed while adjusting for age, sex, age×sex, age^2^, and the first 10 principal components of the genetic information, and summary statistics for the SNPs included in the genetic instrument for adult height were obtained. The above outcome summary statistics were developed using PLINK 2.0 [27].

F statistics, which should be over 10 to avoid weak instrument bias, were 153.7 and 87.0 for the CKDGen and the UK Biobank outcome data, respectively [28].

### Statistical methods for summary-level MR for kidney function traits

Overlapping SNPs with compatible alleles that were not palindromic with intermediate allele frequencies were utilized as the genetic instrument for our summary-level MR investigation.

The main method for summary-level MR was the standard multiplicative random-effect inverse variance weighting method, which can handle variant-specific heterogeneity and is more conservative than the conventional fixed-effect model [29]. Heterogeneity was assessed by Cochran’s Q statistics. To test whether there was a disproportionate effect from a single SNP, single SNP analysis, calculating the causal estimates from a SNP at a time, and leave-one-out analysis, leaving one SNP out of the analysis at a time, were performed.

Causal estimates by the inverse variance weighting method can still be biased by unbalanced pleiotropic effects; thus, additional pleiotropy robust MR sensitivity analysis is necessary to support MR assumptions. The current literature suggests several robust methods and performing a set of analyses, preferably from each domain, is encouraged [30]. First, MR-Egger regression, which yields pleiotropy-robust causal estimates, was conducted [31], with calculation of the MR-Egger intercept P value, which is used to indicate the presence of a significant pleiotropic effect. Another sensitivity analysis was carried out using the weighted median method, which provides valid causal estimates even in the presence of invalid instruments in up to 50% of instrumented weights [32]. Among outlier-robust methods, we used MR-Robust, which entails inverse variance weighting analysis but downweighs outlier effects [33]. We also applied MR-Robust Adjusted Profile Score (RAPS), which provides estimates that are valid when pleiotropic effects are normally distributed at approximately zero [34]. Next, the contamination mixture method, which can detect subgroups of genetic variants with similar causal effects and perform robust MR analysis in the presence of invalid instrumental variables, was implemented [35].

Finally, we performed multivariable MR [36] while adjusting for the effects of BMI, which is one of the most widely recognized anthropometric factors affecting various health outcomes, including the risk of CKD. Multivariable MR has particular strength when genetic variants that are only associated with an exposure trait of interest but not with related confounders are rare, as the method can directly adjust the genetic effects of instrumented SNPs on confounding phenotypes. For multivariable MR to support causal effects, genetic instruments should attain the assumptions as conventional MR analysis. Multivariable MR additionally requires an exclusive association between the instruments and risk factors. As BMI and height are very closely related anthropometric parameters and as the biological effect of BMI is large, we aimed to investigate causal estimates from height independent of BMI by implementing multivariable MR. The effects of BMI were provided by another GWAS meta-analysis undertaken also by the GIANT consortium and focusing on BMI traits [37]. Conditional F statistics for eGFR and CKD outcomes were 18.5 and 16.5, respectively, and information for the input data used for the multivariable MR analysis is presented in S7 and S8 Tables.

The summary-level MR analysis was performed using the TwoSampleMR, Mendelian Randomization, and MVMR packages in R [38, 39]. Bidirectional MR was not carried out because kidney function traits in this study were measured mostly in the middle- to old-age population, whereas height was generally determined before reaching adulthood. Sex-stratified MR analysis was not applied because the CKDGen data do not provide sex-specific summary statistics.

## Results

### Baseline characteristics of the observational dataset

The median age of the UK Biobank participants in the observational analysis dataset was 58 years old, and 45.6% of them were males. The baseline characteristics of the study participants according to adult height above the median (≥ 162 cm for females, ≥ 176 cm for males) for each sex are described in Table 1. Those with a taller height had a younger median age and a lower BMI or proportion of obesity. However, among male participants, central obesity was common in those with a taller height. Those with a height taller than the median also had a higher household income. Regarding comorbidities, those who were taller than the median had a lower proportion of hypertension, dyslipidemia, and diabetes than those shorter than the median.

**Table 1:** Baseline characteristics of the observational analysis dataset in the UK Biobank cohort.

	Female height below the median (<162 cm)	Female height above the median (≥ 162 cm)	Male height below the median (< 176 cm)	Male height above the median (≥ 176 cm)
	(N = 112,114)	(N = 141,332)	(N = 106,497)	(N = 107,239)
Age (years)	59.0 [52.0;64.0]	56.0 [49.0;62.0]	60.0 [52.0;64.0]	57.0 [49.0;63.0]
Body mass index (kg/m^2^)	26.7 [23.9;30.4]	25.6 [23.1;29.1]	27.5 [25.2;30.3]	27.1 [24.8;29.8]
Obesity (≥ 30 kg/m^2^)	30366 (27.1%)	29048 (20.6%)	28732 (27.0%)	25465 (23.8%)
Waist circumference (cm)	83.0 [75.0;92.0]	83.0 [76.0;92.0]	95.0 [88.0;102.0]	97.0 [90.0;104.0]
Central obesity (≥ 102 cm for male ≥ 86 cm for female)	40389 (36.0%)	51451 (36.4%)	28744 (27.0%)	36169 (33.7%)
Weight (kg)	65.8 [58.9;74.9]	71.5 [64.2; 81.1]	79.8 [72.9; 88.1]	88.7 [80.9; 98.1]
Fat free mass (kg)	41.7 [39.2;44.6]	45.5 [42.8;48.7]	59.3 [55.3;63.6]	66.7 [62.4;71.6]
Household income before tax				
< 18000 ₤	27263 (30.5%)	24416 (20.4%)	24178 (26.0%)	14821 (15.2%)
18000 to 30999 ₤	24903 (27.9%)	30182 (25.2%)	24824 (26.7%)	21752 (22.3%)
31000 to 51999 ₤	20977 (23.5%)	32236 (26.9%)	23522 (25.3%)	27515 (28.3%)
52000 to 100000 ₤	13226 (14.8%)	25957 (21.6%)	16646 (17.9%)	25577 (26.3%)
> 100000 ₤	2896 (3.2%)	7187 (6.0%)	3873 (4.2%)	7699 (7.9%)
Hx of angina, heart attack, or stroke	5117 (4.6%)	3517 (2.5%)	11267 (10.6%)	7009 (6.5%)
Moderate physical activity (days/week)	3.0 [2.0; 6.0]	3.0 [2.0; 5.0]	4.0 [2.0; 6.0]	3.0 [2.0; 5.0]
Smoking				
Never smoker	67618 (60.6%)	82814 (58.8%)	50629 (47.8%)	53579 (50.1%)
Ex-smoker	33934 (30.4%)	45602 (32.4%)	41464 (39.1%)	40641 (38.0%)
Current smoker	10013 (9.0%)	12487 (8.9%)	13871 (13.1%)	12665 (11.8%)
Hx of hypertension	24052 (21.6%)	20579 (14.6%)	30152 (28.7%)	22541 (21.2%)
Systolic BP (mmHg)	136.0 [123.5;150.0]	131.0 [119.5;144.5]	140.5 [129.5;153.0]	138.0 [127.5;149.5]
Diastolic BP (mmHg)	81.0 [74.0;87.5]	79.5 [73.5;86.5]	84.0 [77.5;90.5]	83.5 [77.0;90.5]
Hx of dyslipidemia	18120 (16.3%)	14085 (10.0%)	28426 (27.0%)	20639 (19.4%)
Total cholesterol (mmol/L)	5.9 [5.1; 6.7]	5.8 [5.1; 6.5]	5.4 [4.7; 6.2]	5.5 [4.7; 6.2]
Triglycerides (mmol/L)	1.4 [1.0; 2.0]	1.7 [1.2;2.5]	1.7 [1.2; 2.5]	1.7 [1.2;2.4]
LDL cholesterol (mmol/L)	3.6 [3.0; 4.2]	3.5 [3.0; 4.1]	3.4 [2.8; 4.0]	3.5 [2.9; 4.0]
HDL cholesterol (mmol/L)	1.5 [1.3; 1.8]	1.6 [1.3; 1.8]	1.2 [1.1; 1.5]	1.2 [1.1; 1.4]
Hx of diabetes	5104 (4.6%)	4374 (3.1%)	8566 (8.1%)	6254 (5.8%)
Hemoglobin A1c (mmol/L)	35.5 [33.1;38.2]	34.9 [32.5;37.4]	35.6 [33.1;38.5]	35.0 [32.5;37.6]
Testosterone (nmol/L)	1.0 [0.7; 1.4]	1.0 [0.71.4]	11.6 [9.4;14.1]	11.6 [9.5; 14.1]
Uric acid (μmol/L)	268.2 [228.1; 314.6]	260.9 [222.6; 305.1]	349.3 [304.1; 399.3[	350.1 [306.3; 398.4]
eGFR (creatinine, mL/min/1.73 m^2^)	93.0 [82.7;99.8]	93.3 [83.1;100.7]	92.1 [82.4;99.3]	92.8 [83.3;100.2]
< 60 or end-stage kidney disease	2900 (2.6%)	2880 (2.0%)	2935 (2.8%)	2153 (2.0%)
eGFR (cystatin C, mL/min/1.73 m^2^)	88.3 [76.0;101.3]	91.7 [79.1;104.2]	87.4 [75.9;99.5]	89.0 [77.9;100.7]
< 60 or end-stage kidney disease	6298 (5.6%)	5293 (3.7%)	5873 (5.5%)	4378 (4.1%)

BP = blood pressure, LDL = low-density lipoprotein, HDL = high-density lipoprotein, eGFR = estimated glomerular filtration rate, Hx = history.

Categorical variables are presented as numbers (percentage) and continuous variables as medians. [interquartile ranges].

### Cross-sectional observational association between height and eGFR

**Fig 2:**
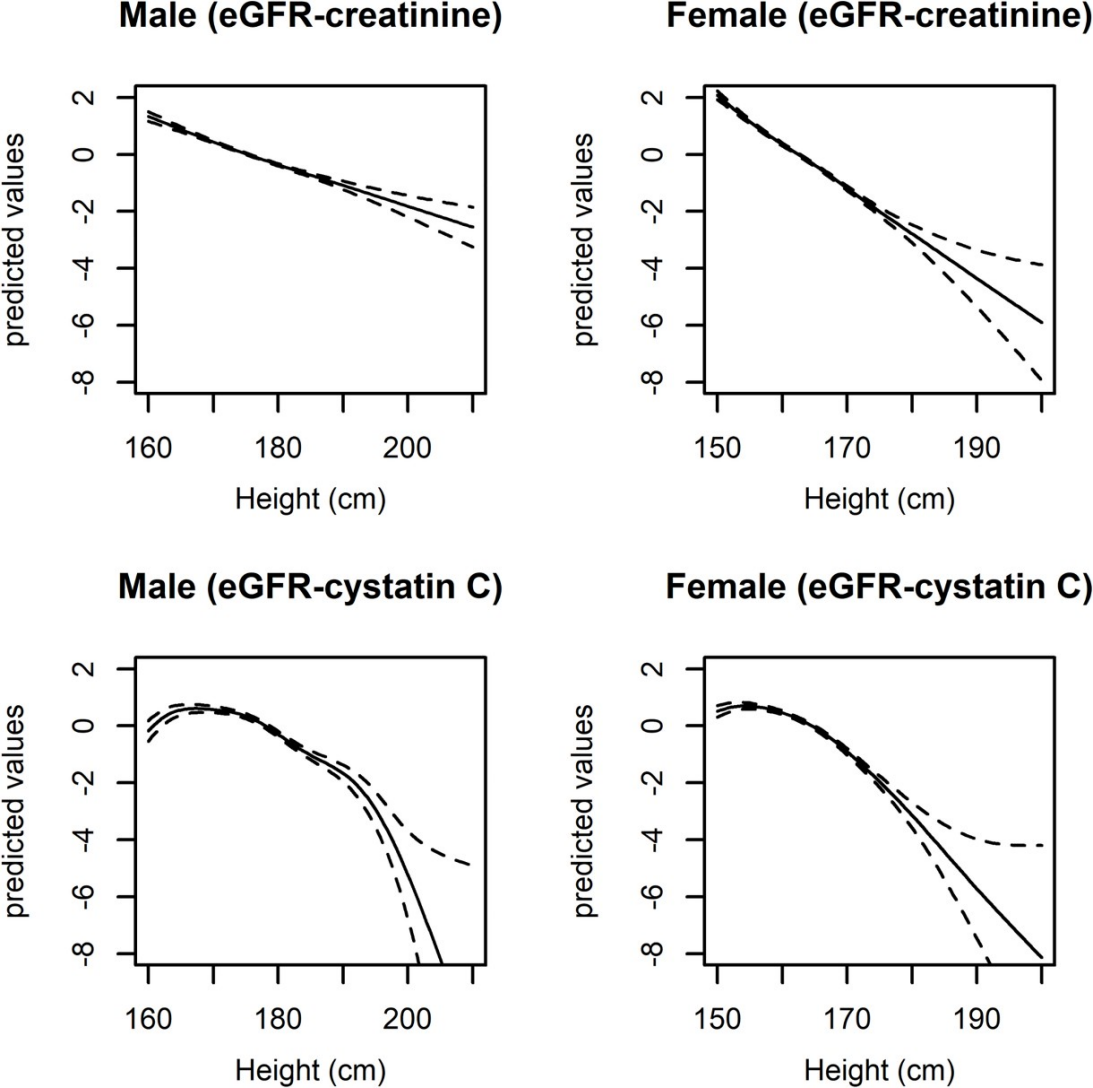
Generalized additive model demonstrating the association between eGFR and height adjusted for age and body mass index for each sex. The x-axes indicate the measured baseline height (cm), and the y-axes indicate zero-centered predictive values for eGFR based on generalized additive models. The broken lines indicate 95% confidence intervals. eGFR = estimated glomerular filtration rate.

Overall, median eGFR values appeared to be higher in those who were taller than the median than in those shorter than the median (Table 1). Additionally, a taller height was generally associated with a lower eGFR, as determined by both cystatin C and creatinine and in both males and females, after adjusting for age and BMI (Fig 2).

In linear regression analysis (Table 2 and S9 Table), a taller height was significantly associated with a lower eGFR, as determined both by creatinine (beta -1.109, SE (standard error) 0.025, P < 0.001) and cystatin C (beta -0.821, SE 0.031, P < 0.001), in the model adjusted for age, sex, and BMI. Furthermore, these results were maintained in subgroups according to median age (age 58 years old) or sex. The inverse association between a taller height and lower eGFR was observed in further adjusted multivariable models, including those adjusted for various anthropometric measurements, metabolic parameters, comorbidities, and social factors. Only the association between eGFR (cystatin C) and height in males in the model adjusted for body shape factors was in disagreement (beta 0.600, SE 0.285, P = 0.035) with the overall trends, but the inverse association as the main result was again noted after adjustment for other characteristics.

**Table 2:** Cross-sectional association between adult height (1 standard deviation increase) and estimated kidney function in the UK Biobank cohort.

Outcome	Subgroup	Multivariable model 1		Multivariable model 2		Multivariable model 3		Multivariable model 4	
		beta (SE)	P	beta (SE)	P	beta (SE)	P	beta (SE)	P
eGFR by creatinine (mL/min/1.73 m^2^)	Total	-1.109 (0.025)	< 0.001	-1.509 (0.103)	< 0.001	-1.039 (0.129)	< 0.001	-1.262 (0.099)	< 0.001
	Male	-0.742 (0.035)	< 0.001	-0.841 (0.224)	< 0.001	-0.963 (0.259)	< 0.001	-0.764 (0.209)	< 0.001
	Female	-1.476 (0.035)	< 0.001	-2.010 (0.185)	< 0.001	-1.439 (0.244)	< 0.001	-1.767 (0.173)	< 0.001
	Age < 58	-1.324 (0.035)	< 0.001	-1.231 (0.141)	< 0.001	-0.994 (0.174)	< 0.001	-1.187 (0.137)	< 0.001
	Age ≥ 58	-0.929 (0.035)	< 0.001	-1.370 (0.151)	< 0.001	-0.811 (0.193)	< 0.001	-0.978 (0.144)	< 0.001
eGFR by cystatin C (mL/min/1.73 m^2^)	Total	-0.821 (0.031)	< 0.001	-2.025 (0.129)	< 0.001	-1.769 (0.161)	< 0.001	-1.671 (0.121)	< 0.001
	Male	-0.727 (0.045)	< 0.001	0.600 (0.285)	0.035	-1.390 (0.328)	< 0.001	-0.754 (0.263)	0.004
	Female	-0.944 (0.043)	< 0.001	-0.731 (0.226)	0.001	-1.472 (0.297)	< 0.001	-1.754 (0.210)	< 0.001
	Age < 58	-1.071 (0.065)	< 0.001	-2.039 (0.184)	< 0.001	-1.670 (0.220)	< 0.001	-1.709 (0.176)	< 0.001
	Age ≥ 58	-0.773 (0.043)	< 0.001	-1.910 (0.181)	< 0.001	-1.798 (0.237)	< 0.001	-1.458 (0.172)	< 0.001

eGFR = estimated glomerular filtration rate, SE = standard error.

Multivariable model 1 = adjusted for age, sex, and body mass index.

Multivariable model 2 = Multivariable model 1 + waist circumference, weight, fat free mass measured by bioimpedance devices.

Multivariable model 3 = Multivariable model 2 + hypertension, systolic BP, diastolic BP, dyslipidemia medication, LDL cholesterol, HDL cholesterol, triglycerides, diabetes mellitus, hemoglobin A1c, testosterone level, uric acid level, history of angina/heart attack/stroke, history of cancer, average moderate physical activity frequency.

Multivariable model 4 was performed with adjustment as multivariable model 3, but multiple imputation by the chained equation method was implemented for missing values.

### Summary-level MR results

**Fig 3:**
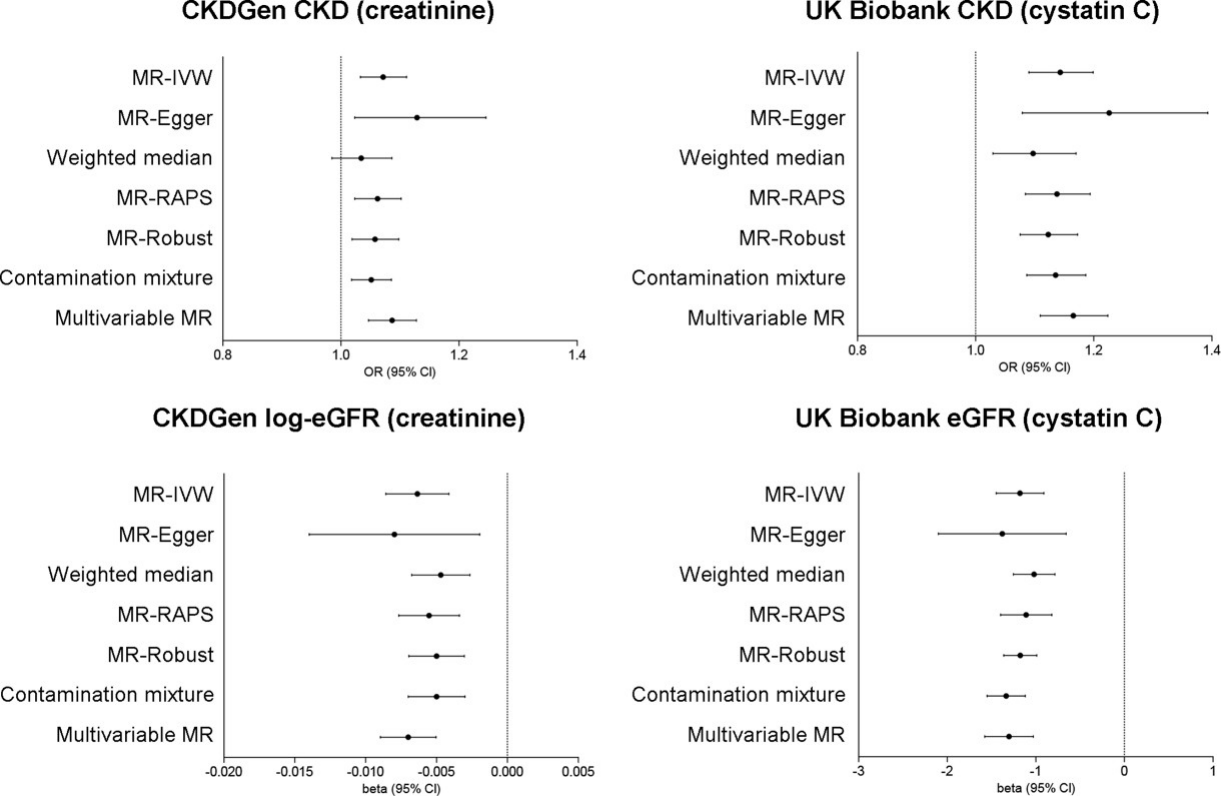
Causal estimates of adult height on kidney function traits in the CKDGen GWAS meta-analysis (N = 567,460) and UK Biobank GWAS (N = 321,405). Kidney function parameters (eGFR and CKD) were based on serum creatinine values in the CKDGen GWAS meta-analysis and on serum cystatin C levels in the UK Biobank GWAS. eGFR was log-scaled in CKDGen data, and raw continuous values were used for UK Biobank data. Multivariable MR was performed with adjustment of the genetic effects of genetic instruments for body mass index. GWAS = genome-wide association study, eGFR = estimated glomerular filtration rate, CKD = chronic kidney disease. MR = Mendelian randomization, MR-IVW = Multiplicative random effect inverse variance weighting, MR-RAPS = Mendelian randomization-robust adjusted profile score, OR = odds ratio, CI = confidence interval.

After disregarding 27 SNPs that were palindromic with intermediate allele frequencies and 5 SNPs that did not overlap with the CKDGen results, 665 SNPs were utilized as the genetic instrument for eGFR, and 666 SNPs were used for CKD (S2 and S3 Tables). Causal estimates for eGFR (creatinine) and CKD (creatinine) indicated that a taller genetically predicted adult height was causally linked to a lower log-eGFR (beta -0.006, SE 0.001, P < 0.001) and a higher risk of CKD (beta 0.069, SE 0.019, P < 0.001) when using the CKDGen dataset and the multiplicative random effect inverse variance method (Fig 3 and Table 3). This result remained significant in pleiotropy-robust MR sensitivity analyses, and the MR-Egger regression test for directional pleiotropy indicated no significant directional pleiotropy in the causal estimates for eGFR (MR-Egger intercept P value = 0.572) and CKD (MR-Egger intercept P value = 0.261). In addition, single-SNP analysis, leave-one-out analysis, and funnel plots visually showed the absence of disproportionate effects from some SNPs (S1, S2, S3 Figs). Although the causal estimates remained significant by the weighted median method for log-eGFR (beta = -0.005, SE = 0.001, P < 0.001), the results were nonsignificant for the CKD outcome (beta = 0.034, SE = 0.025, P = 0.178), with generally similar effect sizes and directions as the main causal estimates. Moreover, the results remained significant in multivariable MR analysis adjusted for the effects of BMI on log-eGFR (beta = -0.007, SE = 0.001, P < 0.001) and CKD (beta = 0.083, SE = 0.019, P < 0.001). The scatter plots by the main MR methods are presented in S4 Fig. The causal estimates were similar when we further excluded SNPs with genome-wide significant associations with diabetes mellitus, hypertension, or obesity.

**Table 3:** Summary-level MR results showing causal estimates of taller height on estimated kidney function outcomes.

Outcome cohort	Outcome phenotype	MR method	Cochran’s Q statistics P value	MR-Egger intercept P value	Total SNPs			After excluding SNPs possibly associated with potential confounders		
					beta	SE	P	beta	SE	P
CKDGen meta-analysis	log-eGFR (creatinine, log-mL/min/1.73 m^2^)	MR-IVW	< 0.001	0.572	-0.006	0.001	< 0.001	-0.006	0.001	< 0.001
		MR Egger			-0.008	0.003	< 0.001	-0.008	0.003	0.006
		Weighted median			-0.005	0.001	< 0.001	-0.005	0.001	< 0.001
		MR-RAPS			-0.006	0.001	< 0.001	-0.005	0.001	< 0.001
		MR-Robust			-0.005	0.001	< 0.001	-0.005	0.001	< 0.001
		Contamination mixture			-0.005	0.001	< 0.001	-0.005	0.001	< 0.001
		multivariable MR ^a^			-0.007	0.001	< 0.001	-0.007	0.001	< 0.001
	CKD (creatinine)	MR-IVW	< 0.001	0.261	0.069	0.019	< 0.001	0.059	0.018	0.001
		MR Egger			0.121	0.050	0.016	0.110	0.050	0.029
		Weighted median			0.034	0.025	0.178	0.028	0.025	0.277
		MR-RAPS			0.060	0.019	0.001	0.051	0.019	0.006
		MR-Robust			0.056	0.019	0.004	0.048	0.019	0.013
		Contamination mixture			0.050	0.016	0.003	0.039	0.019	0.011
		multivariable MR ^a^			0.083	0.019	< 0.001	0.069	0.019	< 0.001
UK Biobank GWAS	eGFR (cystatin C, mL/min/1.73 m^2^)	MR-IVW	< 0.001	0.558	-1.178	0.137	< 0.001	-1.082	0.132	< 0.001
		MR Egger			-1.379	0.368	< 0.001	-1.319	0.354	< 0.001
		Weighted median			-1.019	0.119	< 0.001	-1.030	0.118	< 0.001
		MR-RAPS			-1.109	0.148	< 0.001	-1.066	0.144	< 0.001
		MR-Robust			-1.175	0.095	< 0.001	-1.133	0.130	< 0.001
		Contamination mixture			-1.334	0.110	< 0.001	-1.343	0.107	< 0.001
		multivariable MR ^a^			-1.303	0.140	< 0.001	-1.128	0.137	< 0.001
	CKD (cystatin C)	MR-IVW	< 0.001	0.248	0.134	0.024	< 0.001	0.118	0.024	< 0.001
		MR Egger			0.204	0.065	< 0.001	0.189	0.064	0.003
		Weighted median			0.093	0.033	< 0.001	0.091	0.033	0.005
		MR-RAPS			0.129	0.025	< 0.001	0.122	0.024	< 0.001
		MR-Robust			0.116	0.022	< 0.001	0.115	0.023	< 0.001
		Contamination mixture			0.127	0.022	< 0.001	0.129	0.022	< 0.001
		multivariable MR ^a^			0.153	0.025	< 0.001	0.120	0.025	< 0.001

SE = standard error, MR = Mendelian randomization, SNP = single-nucleotide polymorphism, MR-IVW = multiplicative random-effect inverse variance weighting, MR-RAPS = Mendelian randomization robust adjusted profile score, eGFR = estimated glomerular filtration rate, CKD = chronic kidney disease.

^a^ Multivariable MR analysis was adjusted for the effects of genetic predisposition on body mass index of variants included in the genetic instrument.

These results were reproduced when we introduced the GWAS summary statistics from UK Biobank for eGFR (cystatin C) and CKD (cystatin C). Using 667 SNPs of the genetic instrument that were not palindromic (28 SNPs) or did not overlap (2 SNPs) in the UK Biobank dataset (S4 and S5 Tables), the causal estimates of a taller genetically predicted adult height on a lower eGFR or higher risk of CKD remained significant in all of the analyses, including the causal estimates by multiplicative inverse variance weighting [(eGFR; beta = -1.178, SE = 0.137, P < 0.001), (CKD; beta = 0.134, SE = 0.024, P < 0.001)] and all of the pleiotropy-robust MR sensitivity analyses, including multivariable MR analysis adjusted for the genetic effects of BMI [(eGFR; beta = -1.303, SE = 0.140, P < 0.001), (CKD; beta = 0.153, SE = 0.025, P < 0.001)]. Again, in the UK Biobank data, disproportionate effects from some SNPs were not revealed by single-SNP-analysis, leave-one-out analysis or MR funnel plots (S1, S2, S3 Figs); the MR scatter plot is presented in S4 Fig.

## Discussion

In the current study, which involved MR analysis, we identified a causal linkage between taller height and lower eGFR or higher risk of CKD as defined by eGFR. The observational findings showed that adult height is inversely associated with estimated kidney function, even in the stringently adjusted model considering large number of covariates. The identified observational results are extended to causal inference by our MR analysis. The MR analysis results consistently indicated that a taller adult height causally reduces estimated kidney function in middle- to old-age individuals, independent of the effects of BMI.

Two possible hypotheses can be considered to explain the study results. First, a simple explanation is that as eGFR is still an “estimated” metric of kidney function relying on equations and biomarkers, whereby a taller height may cause a higher serum creatinine or cystatin C value, independent of other factors, with a lower eGFR but not actually decreased kidney function [12]. The significant inverse observational association found even after adjusting for various anthropometric information or the results for eGFR (cystatin C), which is less affected by such bias, may discourage this interpretation [40]. Regardless, as directly measured GFR values were unavailable, the possibility that the explanation is true could not be disregarded. If the explanation is valid, the causal linkage between height and eGFR still raises an important issue about underestimating kidney function when relying on eGFR values in taller individuals, even when cystatin C values are measured. Second, there may be causal effects of height itself on actual kidney function in middle-aged to elderly individuals and not only on estimated kidney function, as the results remained significant for eGFR (cystatin C) and after adjusting for various body size parameters. This hypothesis is supported by the idea that higher height may be linked to the relative overload of single nephrons or a larger glomerular size, similar to that observed with obesity [10, 11, 41, 42]. After reaching an age beyond the 40s, which the human body had not evolved to reach in the past, the kidney may fail to maintain its workload, which would be larger in taller individuals than in shorter individuals, resulting in decreased kidney function. Such glomerular overload has explained the consequences of obesity or diabetes in terms of kidney function, starting with glomerular hyperfiltration and finally leading to impaired kidney function [42, 43]. Thus, an effect on kidney function similar to that of obesity may occur in taller individuals, which is another important factor of body size [11]. However, as a direct mechanistic explanation is impossible based on this MR study, further research is warranted to investigate the biological mechanisms of the possible effects of height on kidney function.

There are several limitations to this study. First, as measured GFR values were absent, directly proving the effects of height on actual kidney function was impossible. Despite efforts to reduce the effects of the difference between estimated and actual kidney function by including cystatin C values, further research is necessary to draw conclusions regarding the mechanisms of the causal effects of adult height on kidney function in middle-aged to elderly individuals. Second, the study was mainly based on individuals of European ancestry; thus, it is unclear whether the findings can be directly applied to other ethnicities. Third, there was some sample overlap between the CKDGen data and the samples of the GWAS used to identify genetic instruments, which may cause bias towards false-positive findings. However, the results from the two-sample MR, using the independent UK Biobank data, were similar to that from the CKDGen data, thus, the issue would not likely to change the findings of the current study. Fourth, the study only addressed adult height and estimated kidney function in those of middle to old age, and the link between height and kidney function in the pediatric population or young adults was beyond the scope of this study. Last, as MR analysis is weak in detecting nonlinear effects, it remains to be determined whether the findings can be applied to an extreme height range (e.g., very low height).

In conclusion, taller adult height causally reduces eGFR, as determined by either creatinine or cystatin C values, in middle-aged to elderly individuals. Further study is necessary to elucidate the mechanism of this causal link, and whether a taller adult height causes glomerular overload leading to impaired kidney function later in life should be investigated.

## Supporting information

S1 Fig Single-SNP analysis forest plots. Individual SNP associations with outcome traits are presented. Outcomes were CKD and log-transformed eGFR determined based on serum creatinine-based eGFR values in CKDGen data and CKD and continuous eGFR determined based on serum cystatin C level in UK Biobank data.
(TIF)

S2 Fig Leave-one-out analysis results. MR results omitting each SNP at a time by the multiplicative random effect inverse variance weighted method are presented. Outcomes were CKD and log-transformed eGFR determined based on serum creatinine-based eGFR values in CKDGen data and CKD and continuous eGFR determined based on serum cystatin C level in UK Biobank data.
(TIF)

S3 Fig Mendelian randomization funnel plots. Funnel plots demonstrate square root precision on the y-axis and causal estimate on the x-axis. Asymmetry on each side of the overall causal estimates suggests the presence of a directional pleiotropic effect.
(TIF)

S4 Fig Mendelian randomization scatter plots. Scatter plots showing the effect of a genetic variant on exposure on the x-axis and on outcome on the y-axis. Scatter plots are a helpful method to identify outliers and the overall distribution of SNP effects along with overall causal estimates.
(TIF)

S1 Table Summary statistics of the genetic instrument for height and CKD (CKDGen) based on serum creatinine values. (XLSX)

S2 Table Summary statistics of the genetic instrument for height and log-transformed eGFR (CKDGen) based on serum creatinine values. (XLSX)

S3 Table Summary statistics of the genetic instrument for height and CKD (UK Biobank) based on serum cystatin C values. (XLSX)

S4 Table Summary statistics of the genetic instrument for height and eGFR (UK Biobank) based on serum cystatin C values. (XLSX)

S5 Table Association study of the genetic instruments with potential confounders in UK Biobank data. (XLSX)

S6 Table Summary statistics implemented for multivariable MR analysis with CKDGen data. (XLSX)

S7 Table Summary statistics implemented for multivariable MR analysis with UK Biobank data. (XLSX)

S8 Table Regression coefficients and adjusted R-squared values in the models constructed for cross-sectional observational analysis. (XLSX)

S9 Table Regression coefficients and adjusted R-squared values in the models constructed for cross-sectional observational analysis. (XLSX)

S1 Methods (PDF)
