# Supplementary figures and images for: Causal linkage between adult height and kidney function: An integrated population-scale observational analysis and Mendelian randomization study
Source: PLoS One. 2021 Jul 29;16(7):e0254649. doi: 10.1371/journal.pone.0254649 (PMC8321232; doi:10.1371/journal.pone.0254649)

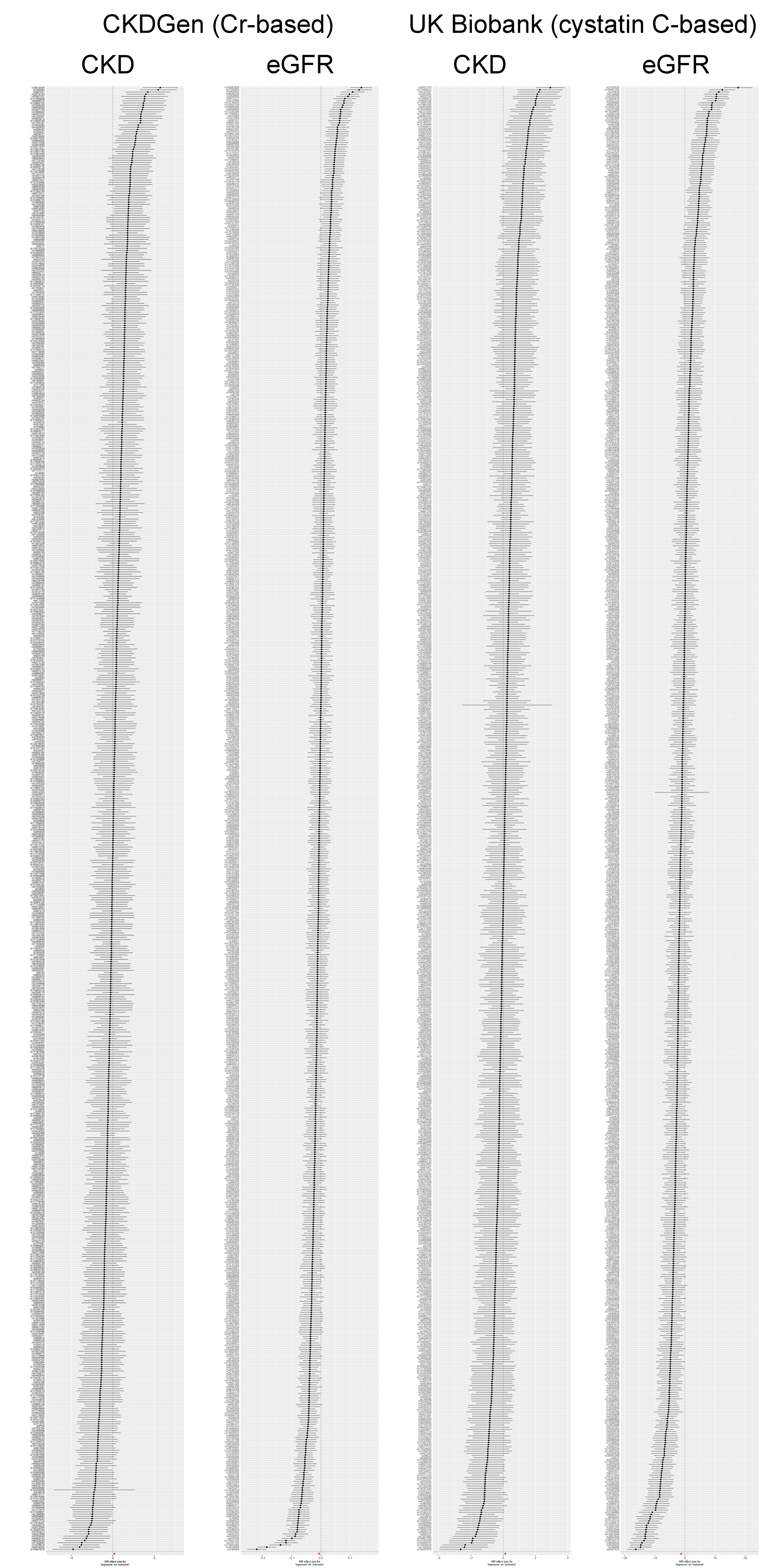

Supplement: S1 Fig — Individual SNP associations with outcome traits are presented. Outcomes were CKD and log-transformed eGFR determined based on serum creatinine-based eGFR values in CKDGen data and CKD and continuous eGFR determined based on serum cystatin C level in UK Biobank data. (TIF) [file pone.0254649.s001.tif]

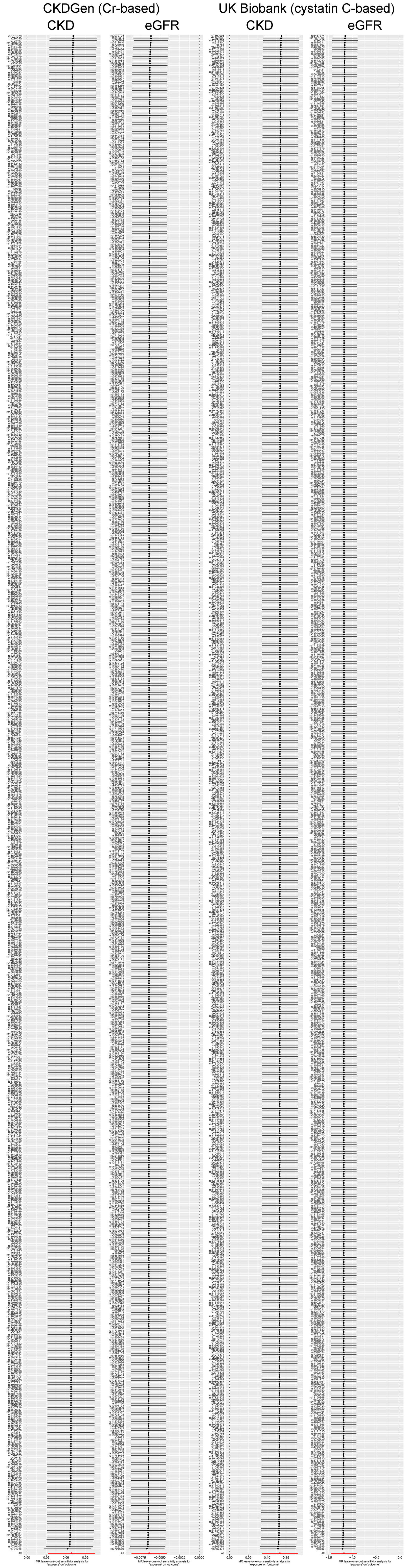

Supplement: S2 Fig — MR results omitting each SNP at a time by the multiplicative random effect inverse variance weighted method are presented. Outcomes were CKD and log-transformed eGFR determined based on serum creatinine-based eGFR values in CKDGen data and CKD and continuous eGFR determined based on serum cystatin C level in UK Biobank data. (TIF) [file pone.0254649.s002.tif]

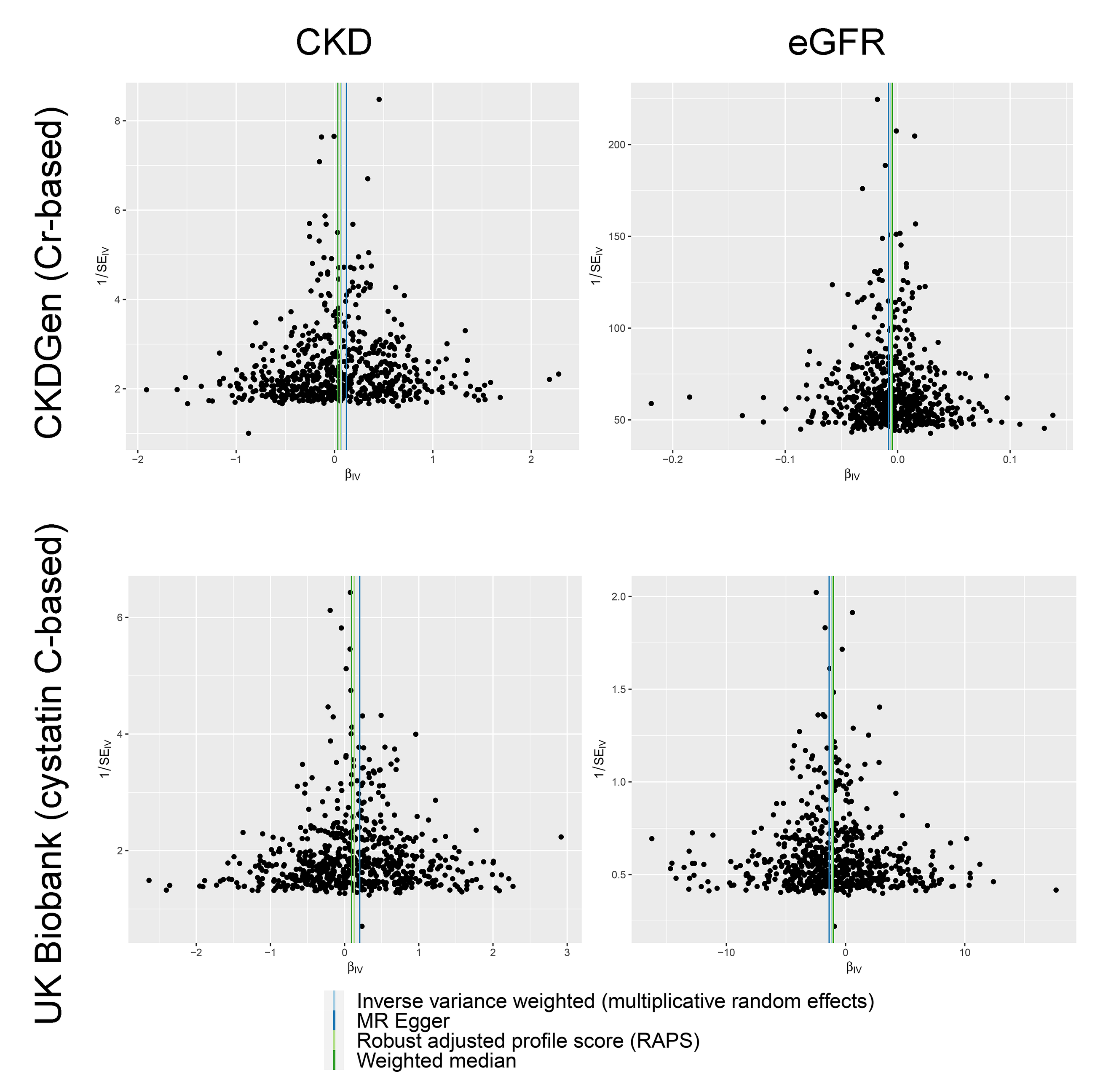

Supplement: S3 Fig — Funnel plots demonstrate square root precision on the y-axis and causal estimate on the x-axis. Asymmetry on each side of the overall causal estimates suggests the presence of a directional pleiotropic effect. (TIF) [file pone.0254649.s003.tif]

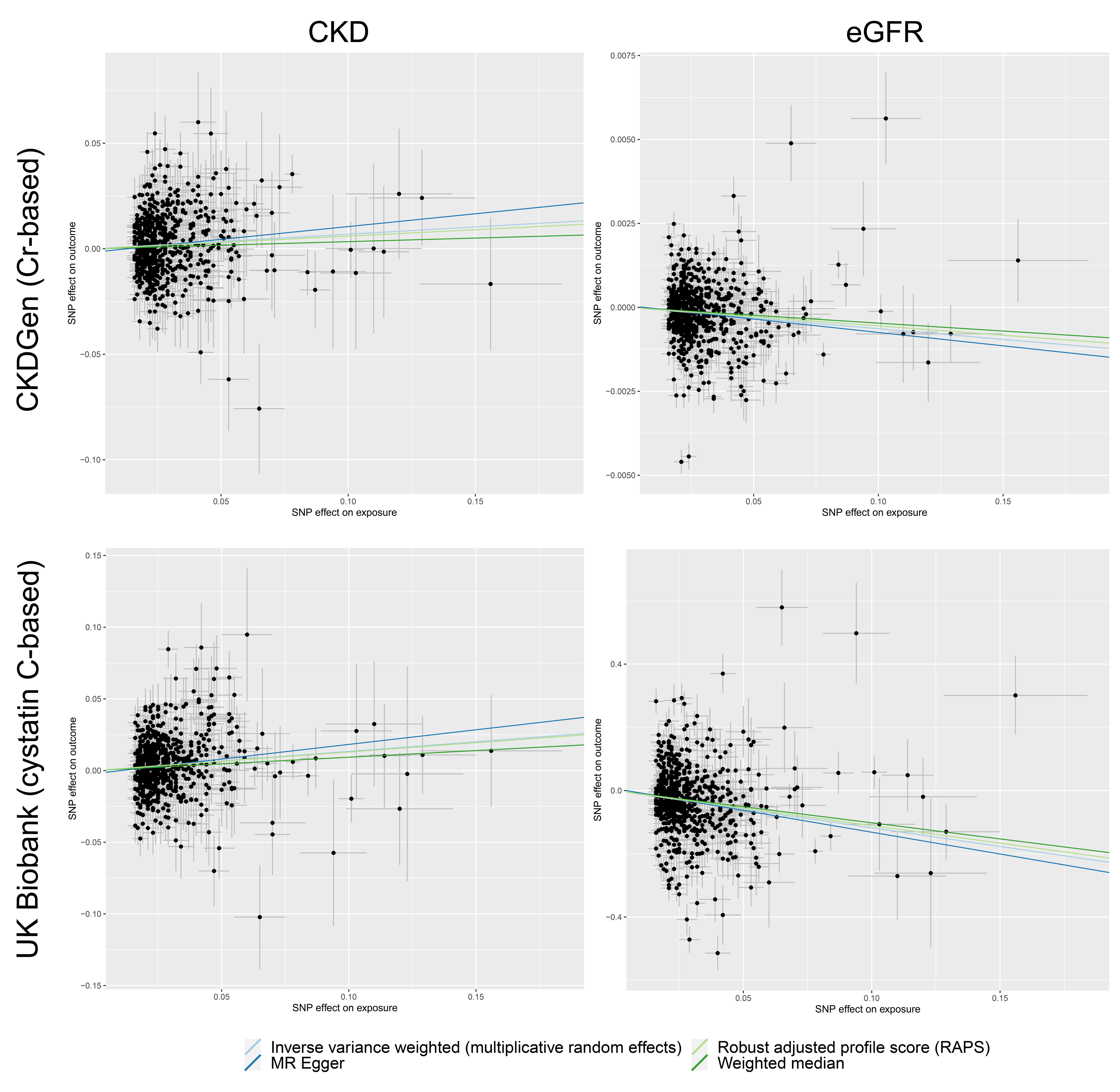

Supplement: S4 Fig — Scatter plots showing the effect of a genetic variant on exposure on the x-axis and on outcome on the y-axis. Scatter plots are a helpful method to identify outliers and the overall distribution of SNP effects along with overall causal estimates. (TIF) [file pone.0254649.s004.tif]
