# Supplementary material for: Causal linkage between adult height and kidney function: An integrated population-scale observational analysis and Mendelian randomization study
Source: PLoS One. 2021 Jul 29;16(7):e0254649. doi: 10.1371/journal.pone.0254649 (PMC8321232; doi:10.1371/journal.pone.0254649)
Supplement: S1 Methods — (PDF) [file pone.0254649.s014.pdf]

S1 Method.

Details regarding the collected covariates

The information for the data from the UK Biobank consortium is available online (URL: https://www.ukbiobank.ac.uk/data-showcase/). The information from the UK Biobank is defined by the field ID.

Height was measured by the staff of the assessment centers (Field ID 12144). Baseline eGFR values were calculated when using creatinine values from the serum creatinine level (Field ID 30700) and ethnicity (Field ID 21000). We also collected the baseline cystatin C level (Field ID 30720) at the baseline visit. The serum creatinine and cystatin C measurements were standardized and obtained by the enzymatic method.

Other information was collected, as follows: age (Field ID 21003), sex (Field ID 31), smoking history (Field ID 20116), body mass index (Field ID 21001, missing N=503), and waist circumference (Field ID 48, missing N=119). Weight (Field ID 21002, missing N=503) and whole-body fat free mass determined by bioimpedance (Field ID 23101) were collected. A history of cardiovascular disease was identified by angina, stroke or heart attack diagnosed by a doctor (Field ID 6150, missing N=1141). Hypertension was determined by a self-reported history of medical treatment for hypertension (Field IDs 6177 and 6153, missing N=3544), and dyslipidemia was identified in the same manner (missing N=3544). A history of diabetes mellitus was determined by self-reporting (Field ID 2443, missing N=1503). Systolic and diastolic BP values were determined by the average of two automated measurements (Field IDs 4080 and 4079), and records with a single missing measurement were considered missing (systolic BP missing N=40,156, diastolic BP missing N=40,144). The hemoglobin A1c level was available from the blood chemistry test results (Field ID 30750, missing N=24,021). Additionally, the total cholesterol (Field ID 30690, missing N=139), LDL cholesterol (Field ID 30780, directly measured, missing N=919), HDL cholesterol (Field ID 30760, missing N=39,543), serum testosterone (Field ID 30850, missing N=44,302), and uric acid (Field ID 30880, missing N=650) levels were collected. Income before tax (Field ID 738, missing N=67,532) was determined as the total household income before tax and collected by touchscreen questionnaire. The frequency of moderate physical activity (10+ minutes) was self-reported (Field ID 884, missing N=24,161).
